# Engineering synthetic microbial communities for soil restoration: from rational design to multi-scale biogeochemical applications

**DOI:** 10.3389/fmicb.2026.1939240

**Published:** 2026-09-22

**Authors:** Mingyi Chen, Yubing Wang, Shouxin Zhang, Shanxu Li, Maorui Zhi, Liya Liang, Kaisi Yang, Xinhua He, Minggang Xu, Lin Yuan

**Affiliations:** 1 College of Food Science and Bioengineering, Tianjin Agricultural University, Tianjin, China; 2 Soil Health Laboratory in Shanxi Province, Institute of Eco-environment and Industrial Technology, Shanxi Agricultural University, Taiyuan, Shanxi, China; 3 School of Biological Sciences, University of Western Australia, Perth, WA, Australia; 4 Shanxi Dongda Soil Technology Co., Ltd., Yangquan, Shanxi, China

**Keywords:** bioremediation, machine learning, nutrient cycling, plant stress tolerance, soil microbiome, synthetic microbial communities

## Abstract

Soil degradation caused by intensive land use, pollution, and climate stress threatens food production and ecosystem function. Single-strain inoculants often perform inconsistently because they do not persist or compete well in resident soil microbiomes. Synthetic microbial communities (SynComs) combine complementary microbial functions in defined consortia. Their performance, however, depends on community composition and environmental context. Multi-omics, high-throughput screening, and artificial intelligence (AI) can reduce the number of strain combinations that need to be tested. Yet the roles of AI and other computational approaches are often described imprecisely. We distinguish four roles: direct AI design, AI-assisted candidate discovery, model-guided design, and prediction-only analysis. This framework links computational methods to the experimental steps needed to construct and validate soil SynComs. We examine applications in nutrient acquisition, carbon cycling, pollutant remediation, disease suppression, and tolerance to drought and salinity. Direct AI design has so far been demonstrated only in a few controlled plant systems and soil microcosms. In most studies, machine learning (ML) is used earlier in the workflow to identify candidates, while cultivation, functional assays, and interaction tests determine the final community composition. To bridge this gap, we propose a staged design-build-test-learn (DBTL) framework, which links model evaluation and community reconstruction with testing in non-sterile soil, strain tracking, safety assessment, and field validation. AI can narrow the experimental search space, but empirical validation remains essential to establish causality, persistence, and transferability across soils and hosts.

## 1 Introduction

Soil degradation driven by intensive agriculture, industrial pollution, and climate change threatens global food security and ecosystem stability (Lal, 2015; Zhang Z. et al., 2026). It is often accompanied by organic matter loss, biodiversity decline, and disrupted nutrient cycling. Together, these changes impair soil multifunctionality and limit the effectiveness of conventional restoration practices (Bünemann et al., 2018). Soil microbiomes have therefore emerged as promising targets for restoring ecosystem functions and improving agroecosystem resilience (Banerjee and van der Heijden, 2023).

Most microbial inoculants currently rely on single strains selected for specific traits, such as nitrogen fixation or phosphate solubilization (Bashan et al., 2014). However, their field performance is often inconsistent. Introduced strains may fail to colonize the soil, be excluded by native microorganisms, or lack the metabolic partners needed for stable establishment (Sessitsch et al., 2019). These limitations reveal a fundamental mismatch between the complexity of soil ecosystems and the reductionist design of many microbial inoculants (Panke-Buisse et al., 2015).

Synthetic microbial communities (SynComs) offer a potential way to address this mismatch. SynComs are deliberately assembled consortia with defined members and intended functions (De Roy et al., 2014). They can distribute metabolic tasks among community members. They may also provide functional redundancy and occupy complementary ecological niches. A meta-analysis conducted in living soils found that microbial consortia generally outperformed single strains, although their effects varied among environmental contexts (Liu X. et al., 2023).

Nevertheless, increasing community complexity does not necessarily improve performance. Antagonistic interactions, resource competition, and incompatible growth requirements can reduce community stability and function (Peixoto et al., 2022; Raaijmakers and Kiers, 2022). SynCom performance therefore depends on both community membership and community structure. Species or strain identity, richness, evenness, and phylogenetic or functional diversity may all influence emergent properties (Busby et al., 2017; Foo et al., 2017; Albright et al., 2022; Chialva et al., 2022; Zhan et al., 2022). The relative importance of these features is likely to vary across microbial communities, soil types, and environmental conditions (Matos et al., 2005; Finkel et al., 2020; Li et al., 2021). Effective SynCom design must therefore identify not only which microorganisms to include, but also how they interact under relevant conditions.

This task remains challenging because microbial interactions are incompletely understood and much of soil microbial diversity remains difficult to cultivate (Steen et al., 2019). Recent advances in multiomics and ML can help infer metabolic potential, reconstruct interaction networks, and prioritize cultivation conditions. However, computational predictions cannot substitute for experimental validation. The Design–Build–Test–Learn (DBTL) cycle provides a useful framework for linking prediction with experimentation. Candidate communities are designed, assembled, tested under relevant conditions, and then refined using the resulting data (Shimizu and Matsuda, 2018).

Here, we review advances in AI and computationally supported SynCom engineering for soil restoration. We first compare screening technologies by the information they provide and the uncertainty they retain. We then examine how computational approaches support strain selection, interaction prediction, community optimization, and iterative validation. Throughout the review, we distinguish experimentally validated applications from theoretical proposals and upstream computational uses. Finally, we connect SynCom design with soil restoration practices and assess the ecological and monitoring constraints that shape field deployment.

## 2 From microbial screening to model-ready strain collections

### 2.1 The era of classical cultivation: phenotype-driven isolation (pre-1980s)

Before the advent of molecular tools, soil microbiology relied on culture-based methods. Researchers used selective media, serial dilution, and metabolic assays such as the Most Probable Number technique to isolate and quantify specific microbes (Waksman, 1922; Alexander, 1965). Culture-based screening provides viable isolates that can be assembled, but medium selection, growth rate, dormancy, and dependence on partner organisms exclude many soil taxa (Amann et al., 1995; Figure 1).

**Figure 1:**
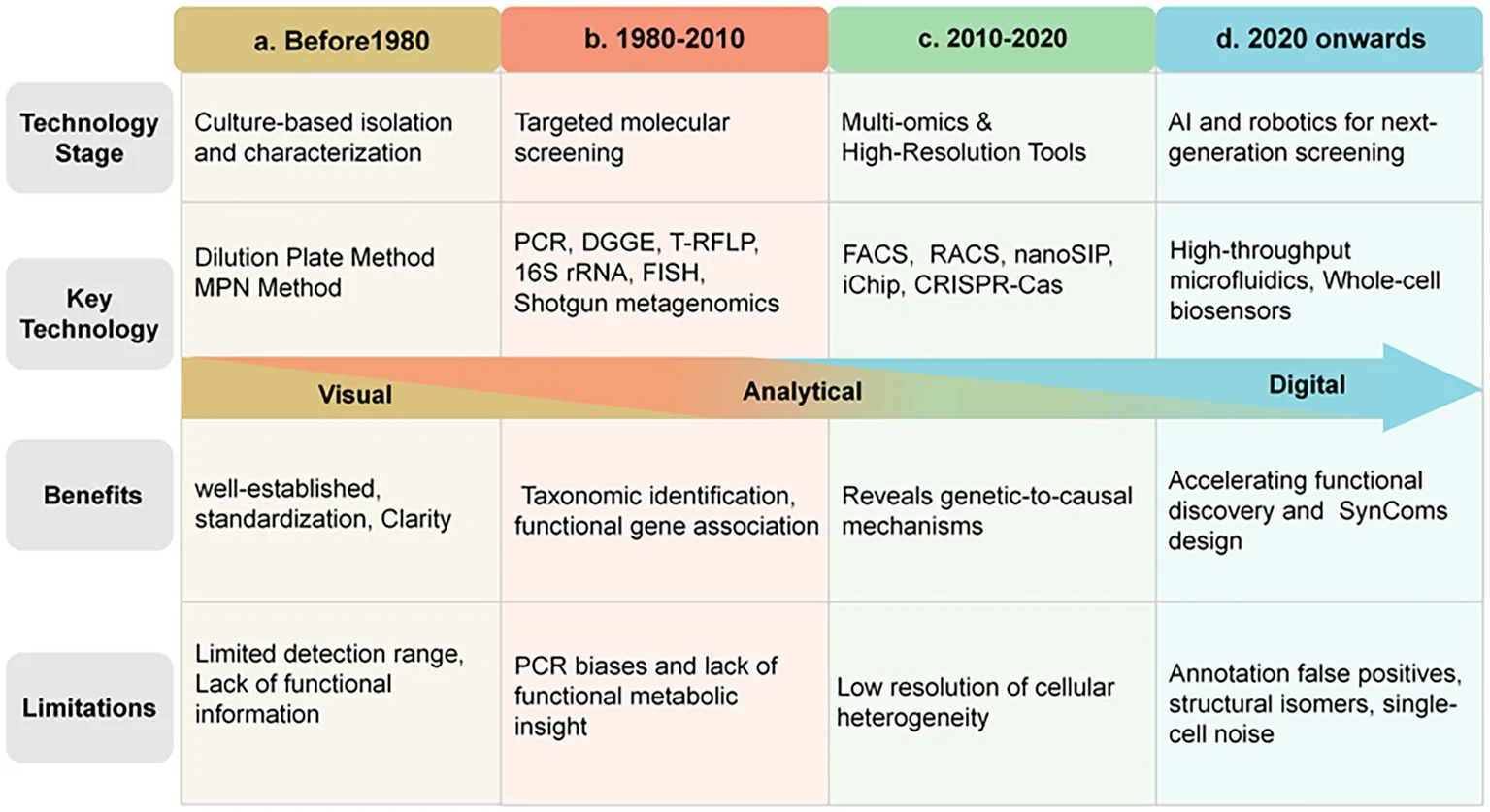
Evolution of soil microbial screening methods. MPN, Most probable number; DGGE, Denaturing gradient gel electrophoresis; T-RFLP, Terminal restriction fragment length polymorphism; FISH, Fluorescence *in situ* hybridization; FACS, Fluorescence-activated cell sorting; RACS, Raman-activated cell sorting; nanoSIP, Nanometer-scale stable isotope probing; iChip, Isolation chip; LightGBM, Light gradient boosting machine.

### 2.2 The molecular revolution: from marker genes to (meta)genomic exploration (1980s–2010s)

To bypass cultivation bottlenecks, the field transitioned toward culture-independent DNA-based profiling. Environmental DNA extraction, PCR, DGGE, T-RFLP, functional-gene amplification, and fluorescence *in situ* hybridization expanded taxonomic and spatial characterization (Amann et al., 1990; Muyzer et al., 1993; Liu et al., 1997). These methods are scalable for candidate discovery, but primer bias, compositional data, and limited strain-level resolution restrict direct inference of community function.

Shotgun metagenomics emerged to profile total environmental DNA without targeted amplification. It can recover genes, pathways, and metagenome-assembled genomes from uncultured taxa (Handelsman et al., 1998; Fierer et al., 2012; Ma et al., 2023). However, metagenomic data describe functional potential rather than expressed activity, and incomplete assemblies or annotations can propagate into downstream interaction networks and metabolic models.

### 2.3 Systems biology paradigm: integrated functional profiling and high-resolution characterization

Systems biology integrates metatranscriptomics, metaproteomics, and metabolomics to show which genes, proteins, and metabolites change under particular environmental conditions (Franzosa et al., 2015; Jansson and Baker, 2016). Bulk measurements can mask cellular heterogeneity and often cannot assign metabolites or proteins to their producers. Single-cell sorting, Raman-based methods, NanoSIMS, and stable-isotope probing improve taxon-function and cross-feeding resolution, but high cost, low throughput, destructive sampling, and limited recovery remain constraints (Song et al., 2016; Hatzenpichler et al., 2020; Pett-Ridge and Weber, 2022). *In situ* cultivation platforms such as the isolation chip can recover rare microorganisms that conventional culture methods often miss. Droplet microfluidics can then test many combinations of cultured strains within a short period (Nichols et al., 2010; Ling et al., 2015; Kehe et al., 2019). CRISPR-Cas editing can test whether a particular gene contributes to a useful microbial trait. However, low editing efficiency and unintended genetic changes may limit its use in environmental microorganisms (Rubin et al., 2022). Together, these methods provide a step-by-step route for SynCom design. Multi-omics identifies possible functions, and cell-based measurements show which microorganisms actually perform them. Cultivation provides live strains, while droplet microfluidics identifies strain combinations that produce the desired effect. CRISPR-Cas editing can then test whether particular genes contribute to that effect. These methods therefore answer different questions and provide the information needed for the next stage of SynCom construction (Figure 2; Table 1).

**Figure 2:**
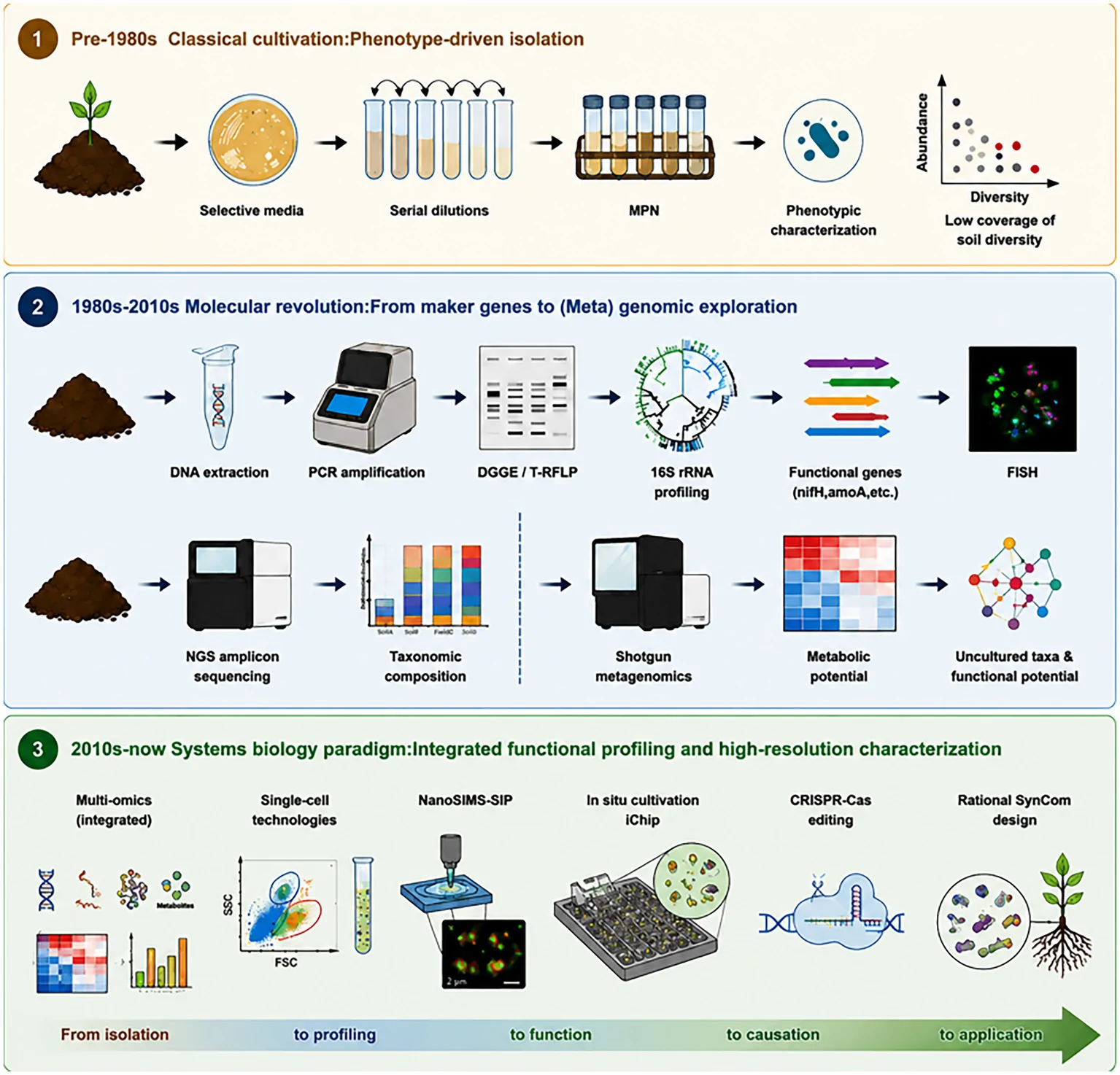
Strategies for expanding the culturable and functional diversity available for SynCom assembly.

**Table 1:** Contributions and limitations of screening technologies for SynCom design.

Technology	Main information	Value for SynCom design	Main limitation	Typical use
Culture and phenotype assays	Viable isolates and measured traits	Produces members that can be assembled and retested	Cultivation and growth-rate bias	Buildable strain library
Amplicon and marker-gene profiling	Taxonomic patterns and selected genes	Enables scalable community comparison	Primer bias, compositional effects, and limited strain resolution	Candidate prioritization
Shotgun metagenomics	Genes, pathways, and metagenome-assembled genomes	Provides broad functional-potential maps	Potential does not equal activity; annotation and assembly errors	Trait matrices and metabolic-model reconstruction
Metatranscriptomics, metaproteomics, and metabolomics	RNA, proteins, and metabolites	Identifies condition-specific activity	Temporal sensitivity and uncertain producer assignment	Pathway and condition prioritization
Stable-isotope probing and single-cell methods	Cell-resolved activity and resource transfer	Links taxa to active functions	Cost, limited throughput, and destructive sampling	Functional and interaction validation
*In situ* cultivation, droplets, and automated phenotyping	Viability and combinatorial performance	Directly tests isolates or communities	Simplified media and host environments	Build-test data generation

### 2.4 AI and computational roles in SynCom prioritization

The screening approaches described above generate high-dimensional data and many possible strain combinations. Different computational methods differ in their assumptions, input data, and outputs. We organize them into three model families and one integration strategy: mechanistic metabolic models, data-driven AI models, ecological dynamical models, and hybrid workflows. These method classes are separate from their roles in SynCom development. The same method may identify candidate strains, prioritize community compositions, assess strain compatibility, or predict environmental outcomes without directly selecting a SynCom (Table 2).

**Table 2:** Roles of AI and computational methods in SynCom development.

Role in the SynCom workflow	Common methods	Main output and purpose	Required experimental validation	Main evidence limitation	Representative references
Direct AI design	Supervised ML; graph neural networks; optimization coupled to a learned model	A ranked set of complete SynComs.	Assemble the predicted communities. Compare high-ranked communities with low-ranked, random, and simple trait-based communities.	Performance within one strain collection; Evidence limited to a small number of controlled systems.	Herrera Paredes et al. (2018), Emmenegger et al. (2023), De la Vega-Camarillo et al. (2025)
AI-assisted candidate discovery	RF and related classifiers; sequence and representation models; metagenomic binning; Co-occurrence networks.	A reduced candidate pool or a ranked list of taxa and functions. The aim is to focus cultivation and functional screening.	Recover or culture candidate strains and verify the predicted function.	Associations do not establish direct interactions or causal functions. Network edges may arise from shared environmental responses.	Weiss et al. (2016), Nissen et al. (2021), Ruan et al. (2024).
Model-guided design	Genome-scale and community metabolic models; flux balance analysis; generalized Lotka–Volterra models; consumer-resource models	Predicted resource use, metabolite exchange, competition, coexistence, or stability.	Test predicted interactions through co-culture, metabolomics, member-dropout experiments, time-series measurements, and community reconstruction.	Predictions depend on genome annotation, gap filling, objective functions, environmental constraints, and model structure.	Xiao et al. (2017), Diener et al. (2020), Ruan et al. (2024), Wang W. et al. (2025), Miao et al. (2026)
Prediction-only analysis	Regression and classification models; neural networks; time-series models; multimodal predictive models	A predicted outcome, risk, or environmental state.	Evaluate the model on data excluded from feature selection, tuning, and training.	Predictive accuracy does not demonstrate SynCom design and microbial causality.	Topçuoğlu et al. (2020), Asnicar et al. (2024)

The role assigned to a study should be determined by the model output and how that output is used, rather than by the algorithm name alone. A single workflow can combine several roles. For example, machine learning may first identify candidate strains, metabolic or ecological models may evaluate compatibility, and an optimization algorithm may then rank complete communities for prospective testing. Co-occurrence analysis alone should be described as computational candidate discovery; it belongs under AI-assisted candidate discovery only when coupled to a learning algorithm.

#### 2.4.1 Mechanistic metabolic models predict resource use and exchange

Mechanistic metabolic models represent biochemical reactions within microbial cells. Genome-scale metabolic models (GEMs) are reconstructed from genome annotations and curated reaction databases. Flux balance analysis predicts feasible or optimal flux distributions under specified objectives and environmental constraints. Community-scale models integrate multiple GEMs to estimate resource overlap, metabolic competition, and potential cross-feeding. These predictions can help prioritize potentially compatible strain combinations (Diener et al., 2020; Ruan et al., 2024; Wang W. et al., 2025).

Metabolic models are computational tools, but they are not AI methods unless coupled with a learning algorithm. Their predictions depend on genome quality, reaction databases, gap-filling procedures, objective functions, and environmental constraints. Different reconstruction methods may therefore yield different predictions for the same community (Hsieh et al., 2024). Predicted metabolic exchanges should be validated through co-culture experiments, metabolite measurements, or growth assays. Member-dropout experiments can further test whether individual strains contribute to community-level functions. Such validation is needed before finalizing SynCom composition or drawing mechanistic conclusions.

#### 2.4.2 Data-driven AI models learn patterns from microbiome data

Data-driven AI models can be divided into three main types: supervised predictive models, representation-learning models, and graph-based models. These model types support different stages of SynCom development.

Supervised models learn associations between microbial features and a predefined outcome. Common methods include random forests (RF), elastic-net regression, and neural networks. Models trained on observational microbiome data can identify taxa, genes, or traits associated with an environmental condition or host phenotype. Their main role is candidate discovery. Models trained on the measured performance of defined communities can instead predict and rank untested community compositions. This approach has been used to predict plant phosphate responses and protection against disease (Herrera Paredes et al., 2018; Emmenegger et al., 2023).

Representation-learning models extract informative features from genomes, metagenomes, and other omics data (Asnicar et al., 2024; Zhou and Zhao, 2025). For example, variational autoencoders can improve metagenomic binning and recover additional microbial genomes (Nissen et al., 2021). These models expand and characterize the candidate pool. However, they do not directly predict SynCom performance unless their features are linked to a downstream predictive model.

Graph-based models represent strains as nodes and their relationships as edges. Graph neural networks can combine this structure with genomic, phenotypic, or ecological features to predict strain compatibility or community performance (De la Vega-Camarillo et al., 2025). Their reliability depends on how the graph is constructed. In particular, co-occurrence edges may reflect shared environmental responses or compositional effects rather than direct interactions (Weiss et al., 2016). Co-occurrence networks can therefore support candidate ranking, but they cannot establish interaction or causality.

The model and training data should match the intended role. Candidate discovery can use strain-level or observational data. Community-level prediction requires performance data from defined strain combinations. In both cases, predictions should be evaluated on independent data and validated experimentally.

#### 2.4.3 Ecological dynamical models evaluate community persistence and stability

Ecological dynamical models describe how microbial populations change over time. Generalized Lotka–Volterra models represent species growth and pairwise effects among community members (van den Berg et al., 2022). Consumer–resource models describe population change through resource uptake, depletion, and competition (Cabal et al., 2020). Depending on their structure, these models can predict persistence, coexistence, local stability, recovery from disturbance, and resistance to invasion.

Their main role in SynCom development is to evaluate the expected dynamics of candidate communities. For example, they can identify combinations in which one strain is predicted to dominate or other members are likely to disappear. They can also test how a community may respond to perturbations or invasion. These predictions can be used to deprioritize potentially unstable combinations before experimental assembly (Xiao et al., 2017; Miao et al., 2026).

Reliable parameter estimation generally requires time-series abundance data collected under conditions relevant to the intended application. Equilibrium or endpoint data may also be used, but they require stronger assumptions and provide less information about transient dynamics. Environmental variables such as soil type, nutrient supply, moisture, and host condition should be included when they are expected to alter growth or interaction parameters.

These models also have important limitations. Pairwise interaction terms may not capture higher-order effects. They may also summarize metabolic processes without representing them explicitly. Predicted stability is therefore conditional on the model structure, parameter estimates, and environmental setting. Candidate communities should still be tested through time-course experiments, perturbation assays, or invasion experiments.

#### 2.4.4 Hybrid workflows integrate prediction, mechanistic evaluation, and discovery

No single model addresses every stage of SynCom development. Hybrid workflows connect complementary methods in a sequence that links candidate discovery to experimental selection.

A typical workflow begins with data-driven models that identify candidate taxa or functional traits. Association analyses may provide additional evidence, although co-occurrence alone should not be interpreted as interaction. Metabolic models can then evaluate resource overlap, competition, and potential cross-feeding. Ecological dynamical models can assess persistence and stability under specified conditions. Finally, optimization algorithms can search the remaining combination space and prioritize communities for experimental testing.

Several studies illustrate parts of this strategy. Ruan et al. (2024) combined RF and differential-abundance analysis with community metabolic modeling. The first stage identified candidate degraders, whereas the metabolic model prioritized potentially compatible combinations. Neural–mechanistic models offer a tighter form of integration by embedding ML within metabolic constraints (Faure et al., 2023). The Genomically and Environmentally Networked Intelligent Assemblies (GENIA) framework combined graph-based prediction with a genetic algorithm to search for pollutant-removing community compositions (De la Vega-Camarillo et al., 2025).

Each component of a hybrid workflow should have a defined input, output, and role (Topçuoğlu et al., 2020). Predictive models estimate biological outcomes, whereas optimization algorithms search for combinations that satisfy specified objectives and constraints. The resulting communities should be compared with simpler selection rules, random combinations, and low-ranked controls. Experimental validation is still required to determine whether the integrated workflow improves SynCom performance.

## 3 Building stable and testable synthetic communities

AI and computational models can rank strains or combinations, but cultivation and reconstruction determine whether the prediction produces a functional community. SynCom assembly must consider the target function, compatibility among members, establishment in the host or soil, and stability after disturbance. This assembly step is therefore the point at which computational prioritization is converted into a testable biological intervention.

### 3.1 Expanding accessible strains and functions

Many soil microorganisms cannot be cultured on standard laboratory media. Others may enter a viable but non-culturable state (Liu J. et al., 2023). Physiological stimulation, alternative cultivation methods, and metagenomic screening can expand the strains and functions available for SynCom development.

First, physiological signals can stimulate the recovery of dormant cells. Resuscitation-promoting factors remodel bacterial cell walls and can promote growth in some soil bacteria (Lennon et al., 2025). Root-derived compounds and siderophores may also improve the recovery of specific organisms.

Second, *in situ* cultivation devices and microfluidic droplets can reproduce selected features of natural habitats. They can also separate slow-growing organisms from strong competitors (Nichols et al., 2010; Ling et al., 2015; Kehe et al., 2019). These methods increase the probability of recovering previously uncultured strains.

Third, metagenomic screening can provide access to useful genes when the source organism cannot be cultured. For example, biosynthetic gene clusters can be identified and expressed in tractable hosts for functional testing (Hover et al., 2018). This approach expands the accessible functional pool rather than directly recovering the original strain.

Recovered strains and heterologously expressed functions may behave differently when introduced into a community. They should therefore undergo the same assembly and validation procedures as conventional isolates. Their value depends on whether they improve community-level function under the intended host and soil conditions.

### 3.2 Bottom-up and top-down assembly

Bottom-up SynComs are assembled from selected isolates. This approach provides precise control over community membership. It also supports member-dropout experiments, inoculum adjustment, and genetic manipulation.

Top-down approaches simplify a natural community while attempting to retain a target function. They preserve more of the original ecological structure. However, they are harder to interpret because some members and interactions may remain unresolved (Xu et al., 2025).

The selection criteria differ between the two approaches. In a bottom-up SynCom, each member should contribute to the target function, community stability, or establishment. This requirement cannot always be evaluated at the individual-member level in a top-down community. Top-down designs should instead be assessed by whether community simplification preserves the target function and key ecological properties.

Functional redundancy and niche overlap should also be distinguished. Limited functional redundancy can protect community performance when one strain declines. In contrast, strong overlap in resource use may increase competition. Phylogenetic distance can provide an initial estimate of ecological difference, but it does not guarantee compatibility. Candidate communities should therefore be tested directly before they advance to application-level experiments (Figure 3).

**Figure 3:**
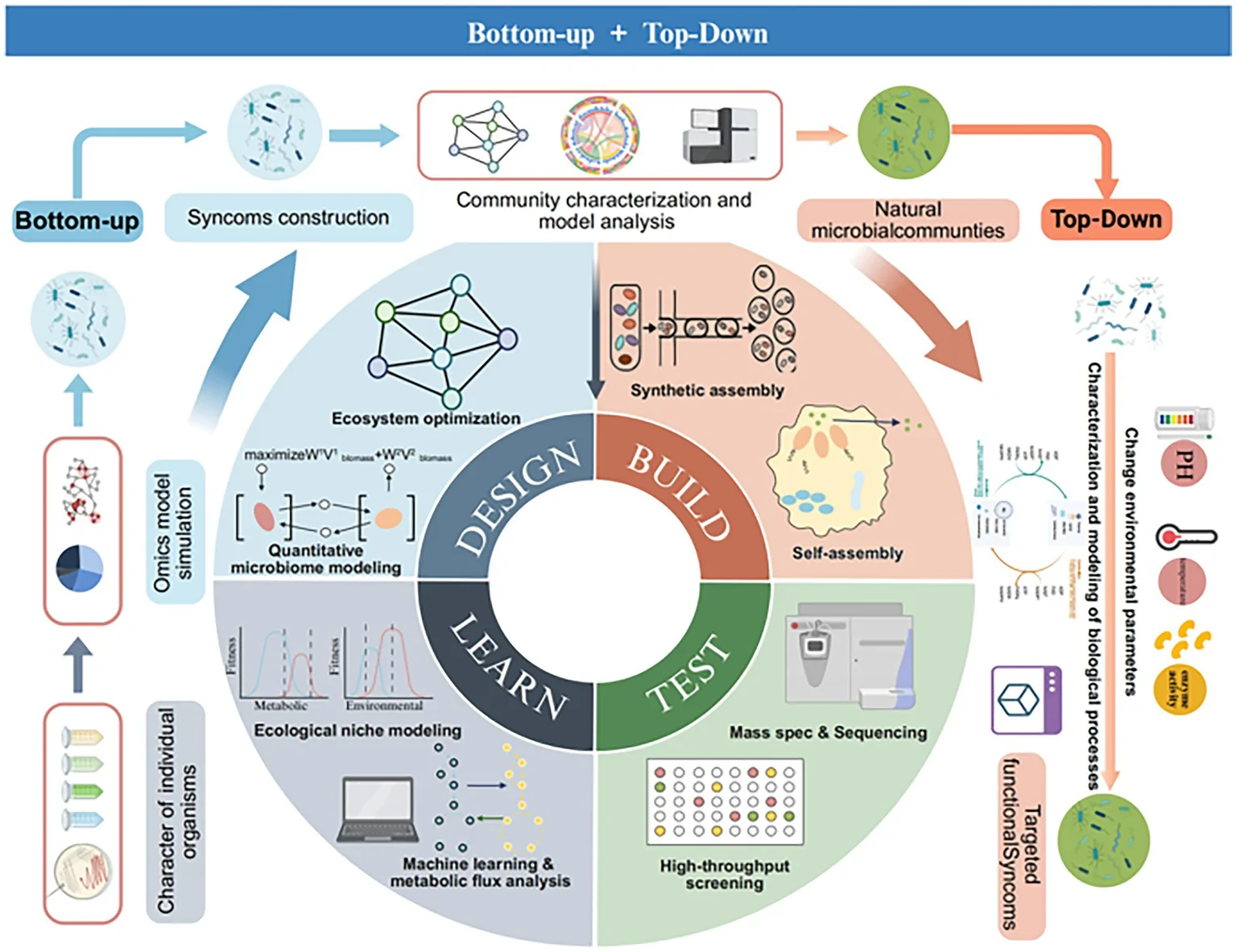
Design-build-test-learn cycle for soil SynCom development.

### 3.3 Compatibility, assembly history, and stability

Community function depends on more than a list of strains. Initial abundance, inoculation order, resource availability, and early colonization can change the final community through priority effects (Carlström et al., 2019). These variables should therefore be controlled or tested during assembly.

Pairwise assays can identify strong antagonism and potential cross-feeding. However, pairwise effects may change in larger communities because of indirect and higher-order interactions. Community-level experiments remain necessary.

Stability should be defined in relation to the design objective. Compositional stability refers to the persistence of community membership or relative abundance. Functional stability refers to the maintenance of the target activity. A community may retain its function despite changes in composition. It may also retain its members while losing function. Both properties should therefore be measured over time.

Relevant tests include serial passage, repeated disturbance, and recovery after drought, rewetting, or nutrient change. Defined bottom-up SynComs can also be evaluated through member-dropout experiments. Invasion assays can test resistance to nonmember strains or background microbiota. Rigorous evaluation further requires appropriate controls, strain tracking, reproducible inoculum preparation, and environmental conditions that reflect the intended application (Zhang Y. et al., 2026).

## 4 AI and computational approaches in SynCom-relevant soil and plant applications

Section 2.4 distinguishes computational methods from their roles in SynCom development. Here, we apply this distinction to soil and plant applications. We classify the approaches according to four questions: What does the model do? How does its output support SynCom development? How is the output tested? What limits the resulting evidence? A single workflow may combine several methods and therefore occupy more than one role (Figure 4; Table 3). The assembly constraints discussed in Section 3 provide the experimental context for interpreting these computational results.

**Figure 4:**
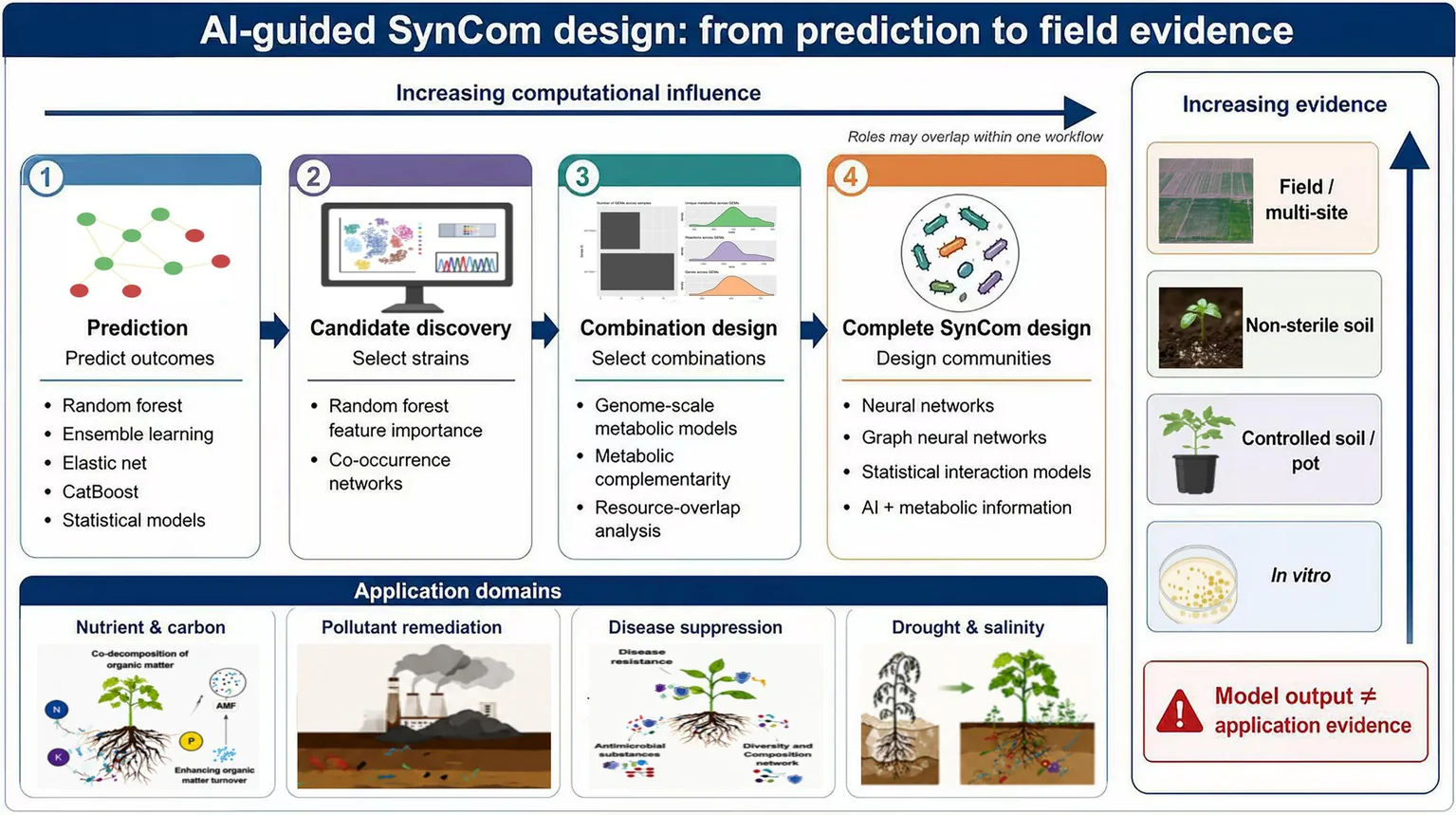
Roles of AI and computational models in SynCom selection and the evidence required for soil and plant applications.

**Table 3:** AI and computational roles in SynCom-relevant soil and plant applications.

Application	Method	Predicted taxa	Evidence classification and boundary	References
Plant phosphate response	Neural network	Effects of untested bacterial combinations on Arabidopsis phosphate traits	Direct AI design; agar-based model, no resident soil community	Herrera Paredes et al. (2018)
Soil carbon cycling	Random forest and network analysis	Taxa associated with carbon content and decomposition properties	Prediction-only analysis; no isolates or SynCom were tested	Zhou et al. (2024)
Community stability and plant growth	Genome-scale metabolic modeling	Compatible combinations based on resource use and exchange	Model-guided design; plant growth rather than carbon storage	Wang W. et al. (2025)
Herbicide remediation	Random forest and co-occurrence network	Herbicide-associated taxa for later culture screening	AI-assisted candidate discovery; assays selected the final community	Zhang et al. (2025)
Organic-pollutant remediation	Random forest, differential abundance, and community metabolic model	Candidate degraders and compatible three-member communities	AI-assisted candidate discovery plus model-guided design; controlled soil tests	Ruan et al. (2024)
Multiple-pollutant remediation	Graph attention network and genetic algorithm	A nine-member community from 45 strains	Direct AI design; limited training records and no matched random-community benchmark	De la Vega-Camarillo et al. (2025)
Heavy-metal treatment	CatBoost and other regression models	Removal efficiency and operating conditions	Prediction-only analysis; wastewater data and no SynCom selection	Xiong et al. (2024)
Bacterial disease suppression	Random forest and elastic-net regression	Protective five-member communities from 35 strains	Direct AI design within a fixed strain pool and gnotobiotic host	Emmenegger et al. (2023)
Fusarium wilt suppression	Random forest and network analysis	Taxa distinguishing field and greenhouse tomato microbiomes	AI-assisted candidate discovery; model did not predict disease or optimize the 205-member community	Zhou et al. (2022)
Drought tolerance	Random forest followed by plant, compatibility, and biofilm assays	Aridity-associated bacteria	AI-assisted candidate discovery; pot scale and short duration	Hao et al. (2026)
Salinity tolerance	Random forest and network analysis followed by functional screening	Salinity-associated and highly connected taxa	AI-assisted candidate discovery; model did not predict the best combination	Li et al. (2025)

### 4.1 Computational approaches play distinct roles in nutrient-related SynCom design and carbon management

Computational approaches play different roles in nutrient-related SynCom design and soil carbon research. Nutrient studies include prospective community design, whereas carbon studies mainly use computational methods for prediction, candidate discovery, or *post hoc* explanation.

Direct AI design has been demonstrated for a defined plant nutrient response. Herrera Paredes et al. (2018) trained a neural network using bacterial communities and *Arabidopsis* phosphate phenotypes. The model proposed previously untested communities expected to alter shoot phosphate accumulation, and most predicted changes were confirmed after community reconstruction. This study therefore connected computational prediction with prospective experimental testing. However, validation was limited to a short agar-based assay, without testing in crops, non-sterile soil, or resident microbiomes.

Mechanistic models provide another route to nutrient-related design. Wang W. et al. (2025) used substrate-use profiles and genome-scale metabolic models to compare all 57 combinations of six beneficial strains. The models estimated resource overlap and potential metabolic exchange. Four- and five-member SynComs with favorable scores remained stable in the tomato rhizosphere and increased plant dry weight by more than 80%. This workflow represents model-guided rather than AI-based design. Validation was limited to sterilized-soil pot experiments, and field-level nutrient-use efficiency was not assessed.

In soil carbon research, computational models more often identify associations than design communities prospectively. Abundance-based analyses may overlook rare but functionally important microorganisms (Bach et al., 2018; Banerjee et al., 2018). Co-occurrence networks can identify highly connected taxa, but their edges may reflect shared environmental responses or compositional effects rather than direct interactions. Network hubs should therefore be treated as putative keystone taxa (Goberna and Verdú, 2022; Berruto and Demirer, 2024). RF and ensemble models are useful for identifying nonlinear associations in complex microbial datasets. However, their feature importance scores do not establish microbial function or causality (Tackmann et al., 2018; Mamet et al., 2019).

The role of these methods depends on when and how they are applied. Zhou et al. (2024) combined ensemble learning with network inference to identify taxa associated with carbon properties across soil aggregates. Because no model-selected community was assembled, the study represents prediction-only analysis. Wang X. et al. (2025) instead used dilution, network analysis, and RF to identify two taxa associated with straw humification. A two-member SynCom increased soil organic carbon by 7.45% in a single-season field experiment. However, the computational analysis selected candidates rather than predicting the performance of their combination. In contrast, Hao et al. (2024) used RF to identify taxa associated with soil multifunctionality in data from a two-year field trial. Because the SynCom was designed before this analysis, the model explained the observed responses rather than guiding community design.

Overall, prospective AI and mechanistic models can prioritize communities when the target phenotype or metabolic criteria are clearly defined. Association-based models are better suited to candidate discovery and *post hoc* interpretation in complex soils. These approaches can be linked sequentially: computational screening identifies candidates, design models prioritize combinations, reconstruction tests their effects, and field trials assess durability. Current evidence does not yet show that a prospectively AI-selected SynCom can produce durable soil carbon gains.

### 4.2 Pollutant remediation provides prospective examples of complete-community selection

Unlike most carbon studies discussed in Section 4.1, several remediation studies prospectively assembled and tested model-selected communities. However, validation remains dominated by culture and microcosm experiments.

Candidate-discovery workflows are common in contaminated soils. Zhang et al. (2025) combined a random forest with a co-occurrence network to identify 16 taxa associated with herbicide residues. Cultivation and degradation assays then reduced this set to four strains. The resulting SynCom removed 60–99% of eight herbicides from black-soil microcosms over 35 days. Computational analysis narrowed the candidate pool, whereas experimental screening determined final membership.

Hybrid workflows can extend candidate discovery through model-guided selection. Ruan et al. (2024) first used RF and differential-abundance analysis to identify potential herbicide degraders. The Super Community Combinations (SuperCC) framework then predicted compatible three-member communities. Reconstructed communities achieved more than 80% degradation in two soil types and were compared with negative communities. This workflow combined candidate discovery with model-guided design. De la Vega-Camarillo et al. (2025) went further by using the GENIA framework to select a nine-member SynCom from 45 genome-characterized strains. GENIA combined graph neural networks with pathway complementarity and redundancy reduction. The selected community removed lignin, atrazine, and perfluorooctane sulfonic acid in culture and retained activity in soil microcosms. However, only one final community was evaluated, limiting evidence for the added value of model-based selection.

Statistical models can also support prospective community selection. Yousef et al. (2026) modeled additive and pairwise effects on bisphenol-A degradation and selected ten previously untested communities. These communities were evaluated in culture and two soil slurries. Culture performance was informative of soil performance, although one soil-condition comparison was not significant. This study supports direct data-driven design rather than necessarily direct AI design. Its main limitations are the small isolate pool and absence of field validation.

Prediction alone does not constitute SynCom design. Xiong et al. (2024) used CatBoost to predict metal removal by sulfate-reducing bacteria and identify influential operating conditions. The model did not select strains or community compositions and therefore represents prediction-only analysis. Moreover, its wastewater-dominated data may not transfer directly to soil SynComs.

Overall, candidate-discovery models narrow the strain pool, whereas mechanistic and data-driven models can select complete combinations for prospective testing. Pollutant remediation provides stronger examples of this progression than the carbon applications in Section 4.1. However, field durability and model-specific benefits remain uncertain. Future studies should compare model-selected communities with random, low-ranked, and function-matched controls. Validation should also distinguish metal immobilization or transformation from removal and assess the fate and ecotoxicity of organic transformation products.

### 4.3 Disease-suppression studies predict community outcomes more often than complete-community design

Computational methods in disease suppression mainly predict outcomes of defined communities or identify candidate protective taxa. Unlike the remediation studies in Section 4.2, the studies discussed here did not prospectively select complete communities from model rankings.

Emmenegger et al. (2023) trained random-forest and elastic-net models on 136 randomly assembled five-member communities. The models predicted *Pseudomonas syringae* colonization in gnotobiotic *Arabidopsis* and were evaluated using 70 new combinations from the same 35-strain pool. Protective strains identified by the models were also tested alone and in combinations. This study provides strong community-level prediction and AI-assisted candidate discovery. However, the validation communities were randomly generated rather than selected from model rankings. The workflow therefore did not directly design protective communities.

Other studies used models to nominate candidates from natural microbiomes. Zhou et al. (2022) used a random forest to distinguish field-grown and greenhouse-grown tomato microbiomes. Network position, field enrichment, cultivation, and antagonism assays then guided isolate selection. Bacterial, fungal, and cross-kingdom SynComs were assembled, with cross-kingdom communities showing the strongest suppression of Fusarium wilt in germ-free plants. However, the model predicted cultivation environment rather than disease suppression or community performance.

Similarly, Mahmoudi et al. (2026) used six years of *Arabidopsis* microbiome data to predict infection status and rank candidate biocontrol organisms. Selected isolates were tested individually, and a protective yeast was later added to a core SynCom. The model therefore supported candidate discovery, whereas final community membership was determined experimentally.

These studies show that the prediction target must match the application claim. Predicting pathogen abundance from a defined community can inform later design, whereas environmental or infection classifiers mainly nominate candidates. Direct design would require prospective selection and comparison of predicted protective and non-protective communities. Validation in crops, non-sterile soils, and field sites would then test whether these predictions transfer beyond controlled systems.

### 4.4 Drought and salinity studies mainly use AI to narrow candidate pools

Recent drought studies combine ML with ecological and functional screening. Hao et al. (2026) used a random forest to identify bacteria with an aridity gradient in *Caragana korshinskii*. Eleven core strains and four strains selected through plant-growth assays formed a 15-member SynCom. Pairwise interaction and biofilm assays then reduced the community to five members. ML narrowed the ecological candidate pool, but experiments determined the final composition. Its main limits are the short pot experiment and the absence of yield, multi-season, or field data.

Li et al. (2025) followed a similar workflow for salinity tolerance. RF identified taxa associated with contrasting *Populus euphratica* rhizospheres, while co-occurrence networks ranked connected taxa. Functional screening retained nine strains, which were used to construct five SynComs. Three communities improved the growth and stress physiology of hybrid poplar. The models nominated candidates but did not predict which community would perform best. Moreover, network associations did not establish direct cooperation among strains.

ML can also play an explanatory role without selecting candidates. Xiang et al. (2025) used a random forest to relate drought-associated plant metabolites to microbiome variation. The four SynCom members were instead identified through deuterium oxide based single-cell Raman sorting and functional assays. The community improved wheat growth under severe drought in controlled pots. Thus, ML explained drought-associated microbiome variation but did not guide SynCom assembly.

Overall, experimental screening remains the main decision point in drought- and salinity-related SynCom development. Current studies support plant-growth effects under controlled stress but provide limited evidence for transfer across soils, crop genotypes, or growing seasons.

## 5 Discussion: SynComs move from prediction to field application

Existing studies range from descriptive analysis to prospective community selection. However, most models are tested within closed strain collections, simplified hosts, short experiments, or soil microcosms. Predictive accuracy in these systems may not translate into field performance.

### 5.1 Model performance does not guarantee application performance

Three types of mismatches commonly weaken application claims. First, the prediction target may differ from the intended function. A classifier of drought state does not directly predict crop growth under drought. Second, training and test data may share the same strains or experimental batch, producing an optimistic estimate of transfer. Third, an effective selected community may not prove that the model improved selection if random, low-ranked, or simple trait-based communities were not tested.

Iterative approaches developed outside soil research may improve efficiency. ML has been used to design defined gut communities and active learning has reduced the number of experiments needed to map microbe-substrate responses (Clark et al., 2021; Connors et al., 2026). These studies do not provide soil application evidence, but they offer transferable experimental strategies. Soil systems will require additional constraints for spatial heterogeneity, resident microbiomes, variable moisture, and host genotype.

### 5.2 Persistence, formulation, and safety

Field deployment requires persistence in non-sterile soil, stable formulation, reproducible production, and acceptable safety. Sterilized substrates can overestimate colonization and plant benefits by removing competitors and releasing nutrients from dead biomass (de Bashan and Nannipieri, 2024). Promising SynComs should therefore be retested in native and contrasting soils. Strain-specific quantitative PCR or sequencing can track persistence, while cultivation and reporter systems can assess viability, activity, or location. Benefits should not be attributed to continued SynCom activity unless introduced members are monitored.

Formulation must preserve member identity, viability, and community composition during storage and application. Alginate, hydrogels, biochar, and encapsulation may improve survival, but unequal storage tolerance can alter member ratios (Pavlicevic et al., 2022; Szopa et al., 2022). Scale-up requires reproducible growth, contamination control, and batch consistency. Safety assessment should address pathogenicity, harmful metabolites, mobile genetic elements, environmental persistence, and non-target effects (Paege et al., 2024; Chemla et al., 2025). Adoption will also depend on cost, storage, handling, and compatibility with farm inputs.

### 5.3 A staged DBTL roadmap

A practical design–build–test–learn workflow should progress through five evidence gates: computational validation, community reconstruction, non-sterile soil testing, product evaluation, and replicated field trials. Each gate should use predefined, stage-specific criteria. Progression should depend on functional performance and ecological stability rather than model accuracy alone.

Results from successful and failed experiments should return to the training dataset. Active learning can then prioritize combinations expected to provide the most information or strongest performance. This approach requires negative results, consistent metadata, and comparable endpoints. The resulting cycle would build cumulative evidence instead of producing isolated demonstrations.

## 6 Conclusion

SynComs offer a structured way to combine microbial functions for soil and plant applications. Controlled studies show that, in selected systems, defined communities can alter nutrient-related plant traits, suppress specific pathogens, degrade selected pollutants, and reduce some effects of drought or salinity. These outcomes depend on the strains, host, soil, and experimental scale. Durable changes in soil structure and consistent transfer across field sites remain poorly supported.

AI and computational models can reduce the number of candidates and combinations that require testing. Direct AI design remains limited to a small number of controlled plant systems and soil microcosms; AI-assisted candidate discovery is more common, model-guided design provides mechanistic assembly rules, and prediction-only analysis does not select communities. Future work should define the prediction target clearly, reconstruct predicted positive and negative communities, track introduced strains in non-sterile soil, and test performance across sites. The central goal is not the most complex model or the richest community, but the simplest community that delivers a reproducible function under realistic conditions.
